# Risk of Band Keratopathy in Patients with End-Stage Renal Disease

**DOI:** 10.1038/srep28675

**Published:** 2016-06-27

**Authors:** Shih-Feng Weng, Ren-Long Jan, Chun Chang, Jhi-Joung Wang, Shih-Bin Su, Chien-Cheng Huang, Sung-Huei Tseng, Yuh-Shin Chang

**Affiliations:** 1 Department of Healthcare Administration and Medical Informatics, Kaohsiung Medical University, Kaohsiung, Taiwan; 2 Department of Pediatrics, Chi Mei Medical Center, Liouying, Tainan, Taiwan; 3 Graduate Institute of Clinical Medicine, National Cheng Kung University, Tainan, Taiwan; 4 Department of Education, University of Taipei, Taipei, Taiwan; 5 Department of Medical Research, Chi Mei Medical Center, Tainan, Taiwan; 6 Department of Anesthesiology, Chi Mei Medical Center, Tainan, Taiwan; 7 Department of Occupational Medicine, Chi Mei Medical Center, Tainan, Taiwan; 8 Department of Leisure, Recreation and Tourism Management, Southern Taiwan University of Science and Technology, Tainan, Taiwan; 9 Department of Child Care and Education, Southern Taiwan University of Science and Technology, Tainan, Taiwan; 10 Department of Ophthalmology, Chi Mei Medical Center, Tainan, Taiwan; 11 Department of Ophthalmology, National Cheng Kung University Hospital, College of Medicine, National Cheng Kung University, Tainan, Taiwan; 12 Graduate Institute of Medical Science, College of Health Science, Chang Jung Christian University, Tainan, Taiwan

## Abstract

This study is a retrospective, nationwide, matched cohort study to investigate the risk of band keratopathy following end-stage renal disease (ESRD). The study cohort included 94,039 ESRD on-dialysis patients identified by the International Classification of Diseases, Ninth Revision, Clinical Modification (ICD-9-CM), code 585 and registered between January 2000 to December 2009 at the Taiwan National Health Insurance Research Database. An age- and sex-matched control group comprised 94,039 patients selected from the Taiwan Longitudinal Health Insurance Database 2000. Information for each patient was collected from the index date until December 2011. In total, 230 ESRD patients and 26 controls had band keratopathy (P < 0.0001) during the follow-up period, indicating a significantly elevated risk of band keratopathy in the ESRD patients compared with controls (incidence rate ratio = 12.21, 95% confidence interval [CI] = 8.14–18.32). After adjustment for potential confounders including sarcoidosis, hyperparathyroidism, iridocyclitis, and phthisis bulbi, ESRD patients were 11.56 times more likely to develop band keratopathy in the full cohort (adjusted HR = 11.56, 95% CI = 7.70–17.35). In conclusion, ESRD increases the risk of band keratopathy. Close interdisciplinary collaboration between nephrologists and ophthalmologists is important to deal with band keratopathy following ESRD and prevent visual acuity impairments.

End-stage renal disease (ESRD) is known as a worldwide public health problem because of its increasing incidence, and the poor prognosis of morbidity and mortality^1^,^2^,^3^. The prevalence and incidence of ESRD have continued to increase worldwide^4^,^5^. Taiwan has the highest incidence and prevalence of ESRD^6^,^7^,^8^.

Band keratopathy is a chronic degenerative condition characterized by the deposition of amorphous, crystalline, grayish to whitish opacities on the corneal surface, most often in the interpalpebral zone^9^. The opacity is caused by sub-epithelial precipitation of calcium hydroxyapatite salt deposition^10^. Because of the deposition and accumulation of calcium, the ocular surface may be disrupted, causing irritation, redness, or photophobia. When the whitish opacity extends to the visual axis, band keratopathy may lead to significant glare and impaired vision. The pathogenesis of band keratopathy primarily involves deposition in the epithelial basement membrane, basal epithelium, and Bowman’s membrane^10^. A variety of factors can cause the sub-epithelial calcium salt deposition, such as elevated serum calcium or serum phosphate levels, hyperparathyroidism, iridocyclitis, phthisis bulbi, and long- standing eye drop use in cases involving ocular hypertension or irritable red eyes^11^.

Elevation of serum phosphate levels is a serious complication and one of the most common problems affecting ESRD patients^12^. Besides elevated serum phosphate levels, secondary renal hyperparathyroidism and the consequent elevated serum calcium levels have long been associated with ESRD^13^,^14^. Additionally, ESRD patients are at a higher risk for increased ocular problems such as increased intraocular pressure^15^,^16^,^17^,^18^ and irritated red eyes^9^,^19^,^20^, which necessitate long-term use of eye drops associated with band keratopathy. Therefore, it is clinically relevant to determine whether ESRD is a predictor of band keratopathy.

Several previous studies have discussed the association between ESRD and red eyes related to calcific deposits in the conjunctivae or corneas^9^,^19^,^20^, but the results of published studies were limited by the small number of patients or the absence of comparative control data. Using a nationwide population-based dataset, we designed a cohort study to investigate the risk of band keratopathy following ESRD in Taiwan. In our cohort study, the ESRD patients were all under dialysis treatment.

## Methods

### Database

On March 1, 1995, a single-payer National Health Insurance (NHI) scheme was launched in Taiwan, which provides extensive medical care coverage for all residents in Taiwan. About 22.60 million individuals (>98%) of the total Taiwanese population of 22.96 million were enrolled in this program as of 2007. The data of our cohort study were obtained from the Taiwan National Health Insurance Research Database (NHIRD). The NHIRD supplies enciphered patient identification numbers as well as information regarding patient birth date, sex, and admission and discharge dates. It also includes the International Classification of Diseases, Ninth Revision, Clinical Modification (ICD-9-CM) diagnoses and procedure codes, prescriptions details, and costs covered and paid by NHI. Ethical approval and informed consent were waived off by the Institutional Review Board of Chi-Mei Medical Center because a public database was used for analysis. Because analysis of datasets in a database does not use identifiable personal information, the requirement of informed consent was waived.

### Study Design

This retrospective, nationwide, matched cohort study involved two groups of participants: a new-onset ESRD group and a matched non-ESRD (control) group.

### Study Participants

Patients and controls were recruited in the period 2000–2009. We included 94,039 ESRD patients who started receiving dialysis treatment after 31 December 2000 and who had received a catastrophic illness certificate with the code number 585 between 1 January 2000 and 31 December 2009. Patients with unknown sex or missing data were excluded. Patients diagnosed as having band keratopathy (ICD-9-CM code 371.43) prior to ESRD were also excluded.

For each ESRD case, one control without ESRD was selected from the longitudinal Health Insurance Database 2000 (LHID2000). LHID2000 was a data subset of the NHIRD that contained entire claim data for one million beneficiaries (4.34% of the total population) systemic-randomly selected in 2000. There was no significant difference in age, sex, and health care costs between this sample group and all national health insurance enrollees. The 94,039 controls were matched by age, sex, and index date. The index date for the ESRD patients was the date of their first dialysis, and the index date for the controls was created by matching the date with the ESRD subject’s index date. Moreover, the controls diagnosed with band keratopathy before the index date were also excluded. Each patient was followed up to determine the incidence of band keratopathy until the end of 2011 or censored because of death.

To distinguish all patients who had developed band keratopathy (ICD-9-CM code 371.43), we tracked every patient from his or her index outpatient visit or hospitalization through December 2011. Demographic data (e.g., age and sex) were recorded. Furthermore, we collected information regarding comorbidities including sarcoidosis (ICD-9-CM code 135), hyperparathyroidism (ICD-9-CM codes 252.0, which excluded secondary hyperparathyroidism due to renal disease.), iridocyclitis (ICD-9-CM code 364.0 to 364.3), and phthisis bulbi or degenerated eye (ICD-9-CM code 360.40, 360.41), because these conditions are critical factors that increase the risk of band keratopathy. In this study, the inclusion criteria for sarcoidosis, hyperparathyroidism, iridocyclitis, and phthisis bulbi were documentation of the condition at least once in the inpatient setting or ≥3 times in the ambulatory setting within 1 year before the initial ESRD on dialysis medical service date.

### Statistical Analysis

SAS 9.4 for Windows (SAS Institute, Inc., Cary, NC, USA) was used in this study. Pearson chi-square test was used to compare the demographic characteristics and comorbid disorders between the ESRD and control groups. The incidence rate was calculated as the number of band keratopathy cases identified during follow-up divided by the total person-years (PY) for each group by age, sex, and select comorbidities. The Poisson regression analysis was performed to calculate the incidence rate ratio (IRR), which demonstrated the comparison in the risk of developing band keratopathy between the ESRD and control groups. The adjusted hazard ratio (HR) for developing band keratopathy was calculated using Cox proportional hazard regression analysis. Cumulative incidence rates for band keratopathy of ESRD were evaluated by Kaplan–Meier analysis, and differences in cumulative-incidence rate curves were analyzed using the log-rank test. Additionally, we subdivided the patients into three age subgroups for further analysis: <50 years, 50–64 years, and ≥65 years. Data are presented as mean ± standard deviation (SD), and 95% confidence intervals (CIs) are provided when applicable. Statistical significance was defined as P < 0.05. These statistical assessments were performed in consultation with a statistical expert.

## Results

### Demographic Data

Between 2000 and 2009, 94,039 ESRD patients and 94,039 controls were recruited after excluding ineligible subjects. Table 1 provides the demographic characteristics and comorbid disorders of ESRD patients and age- and sex-matched controls. The mean age of all participants was 62.22 ± 14.65 years. ESRD patients exhibited a significantly higher prevalence of previously reported comorbidities such as sarcoidosis, hyperparathyroidism, iridocyclitis, and phthisis bulbi, than did the controls. The mean follow-up periods for the ESRD and control patients were 5.70 (SD, 2.89) and 5.73 (SD, 2.88) years, respectively.

### Incidence Rates of Band Keratopathy

During the follow-up period, 256 (256/188,078 [0.14%]) patients developed band keratopathy. A significantly higher proportion of ESRD patients (230/94,039 [0.24%]) than control patients (26/94,039 [0.05%]) developed band keratopathy (Table 2). In addition, there was a significant difference in the incidence of band keratopathy between the groups (ESRD patients = 5.20/10000 PY; control = 0.43/10000 PY), and the IRR between the ESRD and control groups was statistically significant (12.21, 95% CI = 8.14–18.32, P < 0.0001; Table 2).

After the two groups were divided by age, we found that ESRD patients <50 years old had the highest incidence rate (8.37/10000 PY), followed by patients aged 50 to 64 years, and patients ≥65 years old. We found significantly higher IRRs for all ESRD age groups compared with their age-matched controls (Table 2).

Male ESRD patients had a band keratopathy incidence of 5.30/10000 PY, whereas male control patients had a band keratopathy incidence of only 0.27/10000 PY, leading to a significant IRR between male ESRD patients and their controls (IRR = 19.71, 95% CI = 9.62–40.37, P < 0.0001). For female patients, a significant difference was also noted between female ESRD patients and their controls (IRR = 8.89, 95% CI = 5.14–14.60, P < 0.0001; Table 2).

In the ESRD group, the incidence rates of band keratopathy, from the highest to the lowest, were in the order of patients with phthisis bulbi (61.41/10000 PY), iridocyclitis (25.54/10000 PY), and hyperparathyroidism (3.09/10000 PY). However, the IRR for band keratopathy associated with comorbidities could not be determined because no band keratopathy was observed in patients with sarcoidosis, hyperparathyroidism, iridocyclitis, phthisis bulbi in the control group. (Table 2)

Table 3 provides the crude and adjusted HRs for band keratopathy, by cohort, during the follow-up period. After adjusting for age, sex, and select comorbid conditions, ESRD remained an independent risk factor for band keratopathy (adjusted HR = 11.56, 95% CI = 7.70–17.36). The comorbidities that were significant risk factors for band keratopathy in both groups included iridocyclitis (adjusted HR = 4.31, 95% CI = 1.07–17.39, P < 0.05) and phthisis bulbi (adjusted HR = 10.02, 95% CI = 2.48–40.49, P < 0.05) after adjusting for age, sex, and select comorbid conditions.

**Figure 1:**
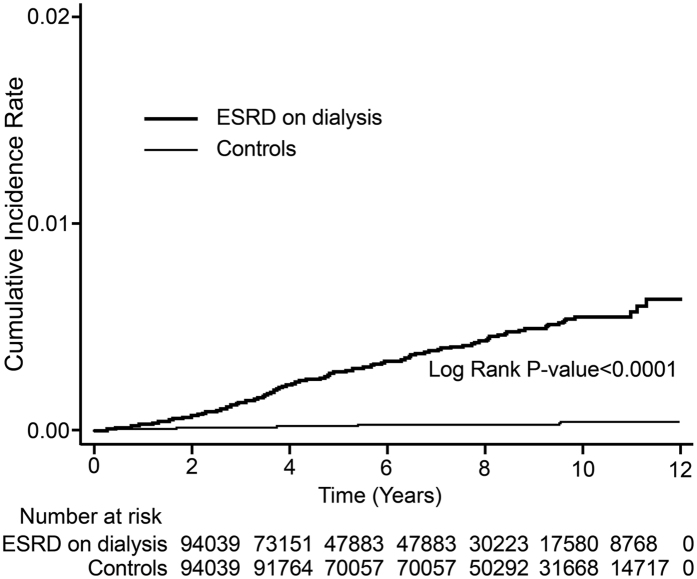
Kaplan–Meier curve of cumulative incidence of band keratopathy in patients with end-stage renal disease (ESRD) and controls during the follow-up period.

The Kaplan–Meier survival analyses revealed higher band keratopathy cumulative incidence rates in the ESRD patients than in the control patients, and the log-rank test was also significant (P < 0.001; Fig. 1).

## Discussion

To the best of our knowledge, our study is the largest-scale population-based study that has been conducted to explore the relationship between ESRD and subsequent band keratopathy. We analyzed 94,039 ESRD patients and 94,039 control subjects. We found that the incidence rate of band keratopathy in ESRD patients was 12.21 times higher than that in controls, and that the relative risk of band keratopathy for patients with ESRD was 11.56 times higher in the full cohort after adjusting for age, sex, sarcoidosis, hyperparathyroidism, iridocyclitis, and phthisis bulbi.

Band keratopathy is a frequent chronic degenerative condition that presents with deposition of grayish to whitish opacities on the corneal surface, most commonly in the interpalpebral zone^11^. These opacities are the result of precipitation of calcium hydroxyapatite crystals in the superficial layers of the cornea, including the epithelial basement membrane, basal epithelium, and Bowman’s membrane^10^. The pathophysiology is multifactorial; besides the well-known chronic ocular conditions such as uveitis and phithsis bulbi, other contributing factors may include elevated serum phosphate levels and increased serum calcium levels that are possibly related to hyperparathyroidism and long-term eye drop use^11^. Although many previous reports drew attention to the link between ESRD and red eyes resulting from calcific deposits in the conjunctivae or corneas^9^,^19^,^20^, there are few studies that directly evaluated the association of band keratopathy and ESRD. Mullaem suggested that band keratopathy, which typically involves amorphous, white, crystalline, and sub-epithelial calcific deposits, is one of the most frequent ocular problems in ESRD patients^21^. Our study is the largest nationwide population-based cohort study to investigate the risk of band keratopathy following ESRD in Taiwan.

Our findings demonstrate an association between band keratopathy and ESRD. The common pathogenic mechanisms for band keratopathy and ESRD include elevated serum phosphate levels, increased serum calcium levels that are possibly related to hyperparathyroidism, and long-term eye drop use in cases involving elevated intraocular pressure or irritated red eyes; these three conditions are discussed separately as follows.

The most well-known pathogenic mechanism common to both conditions is increased serum phosphate and calcium levels. Elevated serum phosphate level is a serious complication affecting ESRD patients receiving hemodialysis^12^. Increased serum calcium levels occur in conjunction with the secondary renal hyperthyroidism^13^,^14^. When the elevated serum phosphate and calcium levels result in calcium phosphate salt precipitation, ESRD patients are susceptible to band keratopathy associated with the deposition of calcium phosphate salts in the form of microcrystalline hydroxyapatite^20^,^21^,^22^. To prevent ongoing hydroxyapatite deposition, elevated levels of phosphate or calcium have to be aggressively controlled.

Another possible pathogenic cause of band keratopathy and ESRD is the greater frequency of long-standing eye drop use in ESRD patients with ocular hypertension and irritable red eyes. There is controversy regarding the effect of hemodialysis on intraocular pressure. Although some studies have demonstrated that intraocular pressure may decrease or remain stable after hemodialysis^23^, most studies describe an increase in IOP during hemodialysis^15^,^16^,^17^,^18^. Various theories about the relationship between elevated IOP and hemodialysis have been postulated, and the most well-accepted theory suggests an influx of volume into the posterior chamber via the ciliary body due to an imbalance in osmolality between the ocular chamber and the blood during hemodialysis^24^. When the intraocular pressure is elevated, ESRD patients usually need long-standing eye drop treatment. Band keratopathy might result from the chronic or excessive use of glaucoma medications with mercury-containing preservatives or the use of pilocarpine, an anti-hypertension agent^11^,^25^. Another condition related to long-standing eye drop use in ESRD patients is the dryness and irritable red eyes related to inflammation of calcific deposition in the conjunctiva^9^,^19^,^20^. Band keratopathy might be associated with the chronic use of symptom-releasing medications manufactured with phosphate-containing preservatives^26^.

We found that the incidence of band keratopathy was greater in younger ESRD patients. ESRD patients aged ≥65 years exhibited the lowest incidence of band keratopathy in the ESRD groups (Table 2), and this was an independent protective factor after adjusting for other confounding factors in both groups (adjusted HR = 0.30, 95% CI = 0.21–0.43, P < 0.05, Table 3). The age-dependent incidence trend was found in the control group. However, paradoxically, the incidence of band keratopathy in ESRD patients aged ≥65 years was the lowest. We have attempted to explain the phenomenon by proposing that the death censoring might play a role in explaining the higher incidence rate in the younger ESRD patients and the low incidence rate in ESRD patients aged ≥65 years. Among elder ESRD populations, there might be a higher proportion of patients who died before band keratopathy development than those in the control group.

Band keratopathy is a common and vision-threatening corneal disorder with a characteristic of deposition with gray to white opacity in the surface of the cornea. Many comorbidities have been associated with band keratopathy, including sarcoidosis, hyperparathyroidism, iridocyclitis, and phthisis bulbi. In this cohort study, we evaluated these comorbidities in ESRD patients and controls and found that iridocyclitis and phthisis bulbi are associated with higher incidences of band keratopathy in the ESRD patients compared with that in the controls (Table 2) and are significant risk factors for band keratopathy in the cohort (Table 3). Many reports demonstrated the link between iridocyclitis and band keratopathy^11^,^27^,^28^. They suggested that although the exact mechanism of calcium-phosphate precipitation in the superficial layers of cornea is unknown, it may result from deposition left as degeneration and necrosis from chronic inflammation related to iridocyclitis^11^,^27^,^28^. ESRD patients with iridocyclitis should be advised to control their inflammation through regular follow-up and treatment by ophthalmologists because of significant association with subsequent band keratopathy.

Band keratopathy in ESRD is an interdisciplinary important issue and close collaboration between nephrologists and ophthalmologists is essential for its management. Nephrologists should be aware of the potential for irritation and visual impairment, which typically presents as white, amorphous, and subepithelial hydroxyapatite crystalline deposition, in ESRD patients under chronic dialysis. The most important concerns for ophthalmologists are evaluating the necessity of treatment in various band keratopathy conditions. Although band keratopathy usually does not impair visual acuity or induce irritation, especially in the early stages, there are some indications for intervention if the condition has progressed. The two major indications for treatment in band keratopathy are decreased vision, which occurs as the calcific precipitation spreads centrally, and mechanical irritation, which occurs because of broken epithelium or a disrupted corneal surface related to calcium accumulation^11^,^21^. Multiple therapies have been attempted for band keratopathy, including mechanical debridement to remove the calcific deposition, EDTA chelation to remove the calcium only and keep the corneal surface smooth^29^,^30^, and excimer laser phototherapeutic keratectomy to remove wide areas of cornea precisely while avoiding trauma to adjacent tissue^31^,^32^,^33^. To avoid additional calcium phosphate deposition, ophthalmologists should maintain caution while prescribing phosphate-containing eye drops to treat irritation^26^, and old mercury-containing eye drops to control the elevated ocular hypertension in ESRD with band keratopathy patients^11^,^25^. Once the diagnosis of band keratopathy is confirmed by an ophthalmologist, prevention of ongoing hydroxyapatite crystalline deposition by aggressive treatment of the increased serum calcium or phosphate levels in ESRD patients on dialysis is of outmost importance for nephrologists. These modalities include dietary recommendations such as substitution of animal protein with vegetarian sources^12^,^34^,^35^, more frequent and more prolonged sessions of dialysis treatments^36^,^37^, dual binder therapy – the use of two phosphorus-binding medications^38^, drug treatment in the patients with secondary renal hyperparathyroidism with agents such as calcitriol analogues^13^ or calcimimetic agents^14^,^39^, and recommending the patients with secondary renal hyperparathyroidism to undergo parathyroidectomy. When dealing with band keratopathy in ESRD patients on dialysis, close cooperation between nephrologists and ophthalmologists is important to reduce the risk of further irritation and visual impairment.

There are several strengths in our study. Based on a nationwide and population-based dataset including a large sample of ESRD patients, the study showed increased precision in risk appraisal and elevated statistical power. In addition, because patients with visual disturbances visit an ophthalmologist rather than a general practitioner in Taiwan, the selection bias in referral centers and chances of misdiagnosis are reduced. Furthermore, this study is a cohort study monitoring the band keratopathy incidence in ESRD and in comparison cohorts with maximum longitudinal data of 10 years. Finally, because sarcoidosis, hyperparathyroidism, iridocyclitis, and phthisis bulbi were taken into account as confounding factors to adjust the hazard ratio of band keratopathy in ESRD patients, our results are reliable.

There are some limitations in our study. Because the sampled patients’ medical history can only be traced back to the year 1996, we cannot confirm that the controls had no ESRD history before January 1996. Additionally, the diagnosis of ESRD, band keratopathy, and other comorbidity disorders relied on ICD-9-codes, which may lead to disease misclassification. Furthermore, some bias may have been introduced because the insurance claims data did not include laboratory data on serum calcium or phosphate levels, or information regarding vitamin D treatment. Besides, intraocular pressure changes after hemodialysis are a controversial topic, since they may increase, remain stable, or decrease. Therefore, one of the mechanisms explaining the increased incidence of band keratopathy based on an increased use of eye drops for the treatment of elevated intraocular pressure in ESRD patients is not very solid. Finally, the evaluation of many comorbidities associated with band keratopathy, including sarcoidosis, hyperparathyroidism, iridocyclitis, and phthisis bulbi in ESRD patients and controls showed that the absence of these comorbidities in the control group compromised the significant incidence ratios of these comorbidities between ESRD and control patients.

In summary, our study showed that after adjusting for age, sex, sarcoidosis, hyperparathyroidism, iridocyclitis, and phthisis bulbi, ESRD patients showed a significantly higher risk of developing band keratopathy during the follow-up period. The association between ESRD and band keratopathy is possible based on the elevated serum phosphate and increased serum calcium levels related to secondary renal hyperparathyroidism, and long-term eye drop use in patients with ocular hypertension or irritable red eyes. We recommend that ophthalmologists should provide adequate treatment modalities in ESRD patients with band keratopathy including observation only, avoidance of phosphate-containing or mercury-containing eye drops, mechanical removal, chelation treatment, and phototheraputic keratectomy. Nephrologists should be aware of the link between the elevated serum phosphate, increased serum calcium levels related to secondary renal hyperparathyroidism and band keratopathy and aggressively control the elevated levels of phosphate or calcium by dietary recommendation, more frequent and more prolonged hemodialysis, dual binder therapy, and medical control (e.g., calcitriol analogues or calcimimetic agents) or recommendations for surgery (e.g., subtotal or total parathyroidectomy) in patients with secondary renal hyperparathyroidism. Close cooperation between nephrologists and ophthalmologists is necessary when dealing with band keratopathy following ESRD and to reduce the irritation and visual impairment development.

## Additional Information

**How to cite this article**: Weng, S.-F. *et al*. Risk of Band Keratopathy in Patients with End-Stage Renal Disease. *Sci. Rep.* **6**, 28675; doi: 10.1038/srep28675 (2016).

## Figures and Tables

**Table 1:** Demographic characteristics and co-morbid disorders in the ESRD and control groups.

	ESRD (N = 94039)	Controls (N = 94039)	*p*-value
	n (%)	n (%)	
Age at index date (mean ± SD)	62.22 ± 14.64	62.22 ± 14.64	1.0000
Age at index date			
<50	18247(19.40)	18247(19.40)	1.0000
50–64	30063(31.97)	30063(31.97)	
≥65	45729(48.63)	45729(48.63)	
Gender			
Male	46597(49.55)	46597(49.55)	1.0000
Female	47442(50.45)	47442(50.45)	
Baseline co-morbidity			
Sarcoidosis	2(0.00)	2(0.00)	1.0000
Hyperparathyroidism	601(0.64)	6(0.01)	<0.0001
Iridocyclitis	185(0.20)	103(0.11)	<0.0001
Phthisis bulbi	75(0.08)	14(0.01)	<0.0001

Note: The demographic characteristics and co-morbid disorders between the ESRD group and control group were compared by Pearson chi-square tests. Abbreviations: ESRD, end−stage renal disease; SD, standard deviation.

**Table 2:** Risk of band keratopathy for the ESRD and control groups.

Characteristics	ESRD				Controls				IRR(95% CI)	*p*-value
	N	Band keratopathy	PY#	Rate^a^	N	Band keratopathy	PY#	Rate^a^		
All	94039	230	442629.66	5.20	94039	26	611089.17	0.43	12.21(8.14–18.32)	<0.0001
Age										
<50	18247	96	114632.79	8.37	18247	3	131483.04	0.23	36.70(11.63–115.82)	<0.0001
50~64	30063	101	150982.85	6.69	30063	9	200383.95	0.45	14.89(7.53–29.45)	<0.0001
≥65	45729	33	177014.02	1.86	45729	14	279222.19	0.50	3.72(1.99–6.95)	<0.0001
Gender										
Male	46597	113	213306.10	5.30	46597	8	297582.70	0.27	19.71(9.62–40.37)	<0.0001
Female	47442	117	229323.57	5.10	47442	18	313506.47	0.57	8.89(5.41–14.60)	<0.0001
Comorbidity										
Sarcoidosis	2	0	9.92	–	2	0	10.07	–	–	
Hyperparathyroidism	601	1	3233.97	3.09	6	0	35.41	–	–	
Iridocyclitis	185	2	783.56	25.54	103	0	618.34	–	–	
Phthisis bulbi	75	2	325.66	61.41	14	0	67.02		–	

Note: The Poisson regression analysis was performed to calculate the incidence rate ratio. Abbreviations: ESRD, end−stage renal disease; IRR, incidence rate ratio; #PY: person-years.

^a^Rate: per 10000 person-years.

**Table 3:** Crude and adjusted hazard ratios of Cox proportional hazard regressions and 95% confidence interval for band keratopathy during the follow-up period for study cohort.

Cohort	Crude Hazard Ratio (95% CI)	Adjusted Hazard Ratio (95% CI)
ESRD		
Yes	12.49(8.32–18.74)*	11.56(7.70–17.36)*
No	1.00	1.00
Age		
<50	1.00	1.00
50~64	0.77(0.59–1.01)	0.83(0.63–1.09)
≥65	0.26(0.18–0.36)*	0.30(0.21–0.43)*
Gender		
Female	1.05(0.82–1.34)	1.09(0.86–1.40)
Male	1.00	1.00
Comorbidity		
Sarcoidosis		
Yes	–	–
No	1.00	1.00
Hyperparathyroidism		
Yes	1.26(0.18–8.96)	0.52(0.07–3.71)
No	1.00	1.00
Iridocyclitis		
Yes	5.88(1.46–23.64)*	4.31(1.07–17.39)*
No	1.00	1.00
Phthisis bulbi		
Yes	21.03(5.23–84.57)*	10.02(2.48–40.49)*
No	1.00	1.00

Note: The adjusted hazard ratio for developing band keratopathy was calculated using the Cox proportional hazard regression analysis. Abbreviations: ESRD, end−stage renal disease.

^*^p-value < 0.05.
